# Using Risk Group Profiles as a Lightweight Qualitative Approach for Intervention Development: An Example of Prevention of Tick Bites and Lyme Disease

**DOI:** 10.2196/resprot.2760

**Published:** 2013-10-30

**Authors:** Desiree Beaujean, Lex van Velsen, Julia EWC van Gemert - Pijnen, Angelique Maat, Jim E van Steenbergen, Rik Crutzen

**Affiliations:** ^1^National Institute for Public Health and the EnvironmentNational Coordination Centre for Outbreak Management (LCI)BilthovenNetherlands; ^2^Department of Psychology, Health and TechnologyUniversity of TwenteEnschedeNetherlands; ^3^Municipal Health OfficeDepartment Infectious DiseasesBredaNetherlands; ^4^School for Public Health and Primary careMaastricht UniversityMaastrichtNetherlands

**Keywords:** ticks, Lyme disease, prevention, audience segmentation

## Abstract

**Background:**

Many public health campaigns use a one-size-fits-all strategy to achieve their desired effect. Public health campaigns for tick bites and Lyme disease (LD) in many countries convey all relevant preventive measures to all members of the public. Although preventing tick bites (eg, by wearing protective clothing or using repellants) and checking for tick bites after visiting a risk area are effective and cost-efficient methods to prevent an individual from contracting a tick-borne disease, public compliance to these methods is low.

**Objective:**

We aimed to identify the group of individuals within the general Dutch population that are at high risk of being bitten by a tick or developing LD and to describe their characteristics, knowledge, and perceptions. The incidence of patients visiting their general practitioner for tick bites and erythema migrans (the first sign of LD) has increased tremendously in the last decades in the Netherlands and other European countries; therefore, our efforts can be used to counter this troubling trend.

**Methods:**

We conducted in-depth semi-structured interviews to identify individuals belonging to the average risk group. Participants were recruited in two ways. Patients who visited two municipal health services travel health clinics (one in a high-endemic area and one in a low-endemic area) were asked to participate. This resulted in 18 interviews. Further, parents were recruited using the convenience sampling method, which resulted in 7 interviews. We discontinued interviewing when the point of data saturation was reached. We analyzed the results immediately after each interview to identify the point of data saturation. Data saturation is when the new interviews provided no new information compared to the previous interviews. The interviews were transcribed and analyzed using inductive thematic analysis.

**Results:**

We identified four groups at risk of being bitten by ticks and developing LD among the general Dutch population. The groups were as follows: (1) outdoor people that check for tick bites, (2) outdoor people that do not check for tick bites, (3) parents that check their children for tick bites, and (4) parents that do not check their children for tick bites. Previous experience with ticks or LD was the main denominator between the groups. Checking for tick bites is a more easily adopted measure than preventing tick bites. Therefore, for all groups, public health efforts in the future should primarily emphasize on the importance of checking for tick bites.

**Conclusions:**

The lightweight qualitative approach presented in this paper is highly relevant in tailoring public health efforts toward specific groups. The profiles of members in each risk group and the motivations underlying the behaviors of the members in each risk group can be used to determine the features and content of a targeted communication strategy about ticks and LD.

## Introduction

Many public health campaigns use a one-size-fits-all strategy to achieve their desired effect. In the context of controlling infectious diseases, a single mode of communication (eg, a leaflet or a television commercial) is often used to convey all relevant evidence-based precautions for a single disease. For example, a Dutch leaflet on preventing food-borne infections advices people to wash their hands with soap before preparing food, after touching raw meat, before eating, and after visiting the toilet or changing diapers; to use a separate cutting board for raw meat and vegetables; to use clean knives for different products; to keep salads and meat cooled during barbeques; not to drink raw milk, etc. Dividing a heterogeneous audience into homogeneous audience segments and subsequently targeting health communication toward these audience segments is a more fruitful approach than distributing a one-size-fits-all message [1]. In the past, many audiences have been successfully segmented for health campaigns such as healthy eating campaigns [2] and promoting physical activity [3]. Segmentation is often based upon demographic, behavioral, and/or psychosocial data and involves the analysis of very large volumes of quantitative data [4].

Lyme disease (LD) is the most common tick-borne disease in the United States and in Europe. In the Netherlands, 564 per 100,000 inhabitants consulted their general practitioner (GP) about tick bites [5]. In 1994, about 39 per 100,000 inhabitants visited their GP for erythema migrans (EM, an associated symptom of LD). This number increased to 134 per 100,000 inhabitants in 2009 [5]. In humans, LD develops in three stages, starting with a circular red skin rash (EM) with fever, headache, fatigue, and depression to a chronic stage that can affect a wide range of body parts, including the brain, nerves, joints, and heart.

Public campaigns in many countries aimed at preventing tick bites and LD use the strategy of providing every member of the public with all the relevant preventive measures [6]. Although preventing tick bites (eg, by wearing protective clothing or using repellants) and checking for tick bites after visiting a risk area are effective and cost-efficient methods to prevent an individual from contracting a tick-borne disease [7], public compliance to these methods is low. According to Marcu et al [8] and Beaujean et al [9], people do not comply with precautions because of the following reasons: people believe that these precautions interfere with how they want to enjoy nature (eg, they refuse to wear long clothes on a hot day), people assume that the risk of tick bites is low, people do not believe that the precautions are effective (eg, they refuse to apply insect repellent products), and people do not know how to identify a tick bite (eg, recognizing and removing a tick). Although it is not impossible to change the knowledge and perceptions of the people about precautions, Mowbray et al [10] recently reported a finding for segmenting the general audience in relation to tick bites and LD. They claim that communication about preventive measures should be tailored toward the knowledge and perceptions of an audience segment about the disease and possible precautions. Thus, it will be more rewarding to provide an audience segment with the advice that they are likely to adopt only. For example, preventive measures that are realistic and fit within the perception of the audience (segment). The measures for preventing LD include checking for tick bites and removing them. Previous studies showed that to match the needs of the target users and to maintain their interest, user involvement is critical in planning and designing the intervention [11,12]. An assessment of mobile-based interventions for management and prevention of human immunodeficiency virus (HIV) infection showed that majority of the interventions failed to attract the attention of their users [13], and users criticized a computer-tailored program for chronic obstructive pulmonary disease (COPD) because the feedback was not tailored to the severity of the disease and could not be used for patients with severe COPD [14]. To develop an intervention that is likely to be used by its target users, it is crucial to gain insight into the perception of the audience segments.

To date, segmentation of the audience has not been performed for providing information about tick bites and LD. In this study, we identified the group of individuals among the general Dutch population that are at high risk of being bitten by ticks or developing LD, and we have described their characteristics, knowledge, and perceptions. The incidence of patients visiting their GP for tick bites and EM has increased tremendously in the last decades in the Netherlands and other European countries [5,15]; therefore, our efforts can be used to counter a troubling trend. Furthermore, to identify audience segments, we will use a lightweight qualitative approach that can be used whenever one does not have access to a large body of information about an audience, or is not in a position to create one, as is often the case in clinical practice. This is especially relevant within the sector of public health, because despite its importance at the societal level, the sector faces critical challenges, including substantial decreases in funding [16,17]. In recent years, public health organizations have been hit hard with cutbacks and layoffs, while more is expected of public health professionals [18,19]. In this study, we take a first step in identifying risk groups on the basis of the previous studies and attempt to understand the differences between the groups based on interview results. In the following sections, we describe the set-up of our methods and present the results for prevention of tick bites and LD. Finally, we discuss the risk groups that we identified and how our method can be used for targeting health communication about the prevention of tick bites and LD.

## Methods

### Overview

The approach of this study consists of two steps: (1) identification of a risk group on the basis of results from previous studies, and (2) in-depth interviews with members of the identified risk group(s) to describe the characteristics of the identified groups. We used a lightweight qualitative approach (small sample of respondents) as a first step in identifying risk groups among the general Dutch population.

### Risk Group Identification

The first step in targeting health communication is to identify groups at high risk for a condition. In the case of tick bites and LD, two high-risk groups are observed among the general Dutch population [20]. The first risk group consists of people that spend a lot of time outdoors (outdoor people), such as hikers, campers, and dog owners. The number of hours spent outdoors per week is related to the risk of tick bites; the greater the number of hours spent outdoors, the higher the incidence of tick bites [21,22]. The second risk group includes children aged from 5 to19 years because of their increased contact with tick habitats, for example, because they play outside [5,23,24]. Since parents are responsible for the health and tick checks of their children, we interviewed parents about their children and tick bites and LD.

### Profiling Risk Group Members: In-Depth Interviews

To profile the members belonging to an average risk group, conducting in-depth semi-structured interviews is an effective approach. These interviews allow for exploring a range of topics and subsequently pursuing a topic in-depth when it appears to be important [25]. This method was also suggested by Mowbray et al [9] to create a basis for designing targeted interventions against tick bites and LD.

Participants were recruited in two ways. People who visited two municipal health services (MHS) travel health clinics (one in a high-endemic area and one in a low-endemic area) for traveler’s vaccination were asked to participate in an interview by a nurse in the infectious disease control department. We opted for this group, because they often recreate outdoors. This resulted in 15 interviews with people who spend time outdoors and 3 with parents. Further, we recruited parents via a convenience sampling method, which resulted in 7 interviews.

We created an interview scheme based on an overview of citizen characteristics that need to be taken into account when developing health interventions [26]. The interview scheme addressed (1) demographics, (2) frequency of visits to high-risk areas, (3) knowledge of ticks and LD (ie, using five statements about recognition of ticks, tick habitats, mode of transmission, and symptoms of LD), (4) experience with ticks and LD, (5) perception and behavior about LD prevention measures (eg, “How severe do you perceive LD?” and “What would you do in the case of a tick bite?”), and (6) tick- and LD-related information seeking behavior (eg, “Where would you seek for information on ticks and LD?”). Interview schemes can be found in Multimedia Appendix 1. An experienced qualitative researcher conducted all the interviews.

Each interview started with a short introduction of its goal, after which the interviewees were guaranteed anonymity. Then, the interviewees provided informed consent and permission for audio recording. Subsequently, the interviewees received a gift voucher as an incentive.

We stopped the interview when all pre-determined themes were discussed, and the interviewee added no new themes. We analyzed the results immediately after each interview to identify the point of data saturation. Data saturation occurred when the interviewer concluded that, compared to the previous interviews, the new interviews provided no new information.

### Analyses

The interviews were transcribed and analyzed using inductive thematic analysis according to the six steps suggested by Braun and Clarke [27]. Inductive thematic analysis focuses on identification and description of themes both implicit and explicit ideas within the interview data. An experienced analyst of qualitative data analyzed the interviews. Step 1 was familiarizing with the data. This involved transcribing the data and reading and re-reading the data in an active manner; searching for meanings, patterns, and writing down initial ideas. This phase was time consuming, but is the bedrock for the rest of the analysis. The formal coding process began after completion of this step. Step 2 was generating initial codes from the data. Codes identify a feature of the data (semantic content or latent) that appears interesting for the analysis and refer to “the most basic element of the raw information that can be assessed in a meaningful manner about the phenomenon.” In this phase, it was important to ensure that all actual information was coded. Step 3 began when all information was coded and a long list of the different codes was identified across the data set. Now, the codes require to be ordered into potential themes. Step 4 included reviewing and refining the themes. Some themes collapsed into each other and some themes were not really themes (the data were too diverse). At the end of this step, we had a good idea of what the different themes were, how they fit together, and the overall story they told about the data. In step 5, we defined and named the themes. Finally, step 6 involved the final analysis and writing up of the results. We will provide an example to make this process more transparent. One interviewee said when we asked him how quickly a tick that has bitten should be removed from the body “I don't know; I only know that it is in its saliva. So, you should never use detergent or alcohol. Because if it has contaminated saliva, it will spit.” A second interviewee said, “I don’t know. I don’t think it matters because either the beast has Lyme or it does not.” Finally, a third interviewee said, “I think directly.” Initially, the interview segments were coded as “knowledge about how soon to remove ticks.” Next, the first two interviewees were coded as “not knowing how quickly to remove a tick”, and the third response was coded as “knowing when to remove a tick.” An overview was finally made when all interviewees were coded according to whether or not they knew how soon to remove a tick.

## Results

### Profiles of Risk Group Members

The responses of the interviewees to the open question about checking for ticks led us primarily to divide both risk groups into the following two subgroups:

1. Outdoor people that do not care about being bitten by a tick and the risks involved (those that do not check) and people that do care and therefore check for ticks when they have visited a high-endemic area (those that check).
2. Parents that check their children for tick bites, and those that do not.

Each subgroup had its own view toward ticks, LD, and preventive behavior, and therefore, we analyzed their responses separately.

Outdoor people that do not check (“I never check for ticks after being outdoors”) constituted a large group (men: 7/14, 50% and women: 7/14, 50%; mean age 43 years). People in this group are often in their backyards and frequently visit a forest. The group of those that check (“I sometimes or always check for ticks after being outdoors”) is relatively small (women: 3/3, 100%; mean age 42 years), and people belonging to this group spend a lot of time in their backyard and can frequently be found in forests, heathland, dunes, and city parks.

Parents that check (“I sometimes or always check my child/children for ticks after they have been outdoors”; men: 2/8, 25% and women: 6/8, 75%; mean age 41 years) accounted for the majority of parents. Their children very often play in the backyards and some of the children of these parents regularly play in forests. Parents that do not check (“I never check my child/children after they have been outdoors”; men: 1/2, 50% and women: 1/2 50%; mean age 42 years) were a small group, whose children often play in the backyards.

### Outdoor People

#### Overview

The main characteristics of the two subgroups within the outdoor people are shown in Table 1. The results indicate that experience with tick bites or LD is the great denominator between the groups and the main reason for a person to shift from checking to not checking for tick bites.

#### Knowledge of Ticks and Lyme Disease

Outdoor people that do not check for tick bites had a widespread knowledge of ticks and LD. Most of them knew that a tick is a little animal. Almost all the participants thought they could get a tick bite in a forest and falsely believed that ticks let themselves fall from trees. About half of our participants also said that ticks can be found in high grass or shrubs. Only few people knew that ticks live in dunes.

I would say only in the forest. And, when you stand under a tree, that’s what you hear often, that they fall out of the tree. That’s all I know.

Woman; age, 18 years

Most interviewees did not think that a tick bite always resulted in LD. Some thought there were individual differences in terms of susceptibility. Most participants thought that a tick should be removed as soon as possible. When asked how they could know whether they have LD, about half of the people mentioned “the red spot” (referring to EM). The remaining participants had no idea. Participants who do check for tick bites had medium to high knowledge of ticks and LD. They knew the size of the ticks, but were not completely informed of their habitat, because they mainly mentioned grassland and forests, and the possibility of ticks falling from trees. According to this group, whether or not a tick bite leads to LD is dependent on the tick being infected or not or how long it is attached to the skin. Finally, they mentioned “the red spot” as a first sign of LD.

#### Experience With Ticks

Several outdoor people that do not check for tick bites told us they had seen a tick mostly on pets. On the other hand, those who checked for tick bites had direct or indirect experience with ticks or LD; they had pets with ticks, friends with LD, or experienced a tick bite followed by an EM.

#### Dealing With Tick Bites

About half of the people that “do not care” would remove a tick using (tick) pliers themselves. However, some erroneous strategies were mentioned, such as burning it off with a cigarette, waiting for the tick to grow big so it can be removed more easily, and twisting the tick when removing it. Some people foresaw negative consequences of using tick pliers, like pain, difficulty in removing the tick, not removing the tick’s head, and finally, the unpleasant feeling of “operating” on yourself. The other half would go to their GP after encountering a tick bite, mostly because they thought they were unable to properly remove a tick by themselves. Participants who check were more confident about their abilities and said they would remove ticks themselves when bitten using (tick) pliers.

#### Preventive Measures

Participants of the group that do not check did not take preventive measures against tick bites when visiting a high-endemic area. They regard staying on paths and using an insect repellent spray on their skin as a viable option to guard themselves against ticks. Several of these people did indicate, however, that if something caught their interest in the forest, they would stray from the paths. Wearing clothes that cover the body was not a viable option according to the respondents as it was uncomfortable or “looks stupid.” After visiting a high-endemic area, people that “did not care” never checked for tick bites. They did not know that they were in a high-endemic area (like dunes), forgot to check, or expected a tick bite to itch. Those that check thought that staying on the paths in the forest could be a viable preventive measure, but they also indicated that they would not comply whenever they wanted to explore the forest off the beaten path. Normally, these participants did not consider wearing clothes that cover the body as a good preventive measure, especially in warm weather. However, the participants also indicated that when the risk of being bitten is high (eg, when being in a high-endemic area), they would comply with this measure.

When it’s 35°C outside, you walk in short pants anyway. And then, you check yourself properly in the evening. But, when the number of ticks runs out of hand, you do put on long thin pants.

Woman; age, 53 years

Finally, for most interviewees, using an insect repellent skin spray was a reasonable measure; it allowed them to wear short pants. All of those that check, stated they checked themselves for tick bites when they were outdoors.

#### Information Seeking Behavior

Outdoor people that do not check for tick bites indicated they would consult a wide range of sources when seeking information on ticks or LD. Most popular resources when searching for information on how to prevent tick bites are the Internet and the MHS, or when bitten by a tick, their GP, the Internet, or the MHS. When searching on the Internet, practically all interviewees would start with a Google search. Those that check expected their MHS to provide them with information on ticks and LD. When searching for information on how to prevent tick bites, they mentioned pharmacies and an online Google search. For information on how to remove ticks, they would resort to their GP or the Internet (with no specific website in mind).

**Table 1:** Characteristics of outdoor people.

	Those that do not check	Those that check
Age	widespread	widespread
Family situation	either living alone, living together without children, or living together with grown-up children	either living alone, living together without children, or living together with grown-up children
Education	widespread	widespread
Pet ownership	about half of the people has a pet	about half of the people has a pet
Direct experience with ticks / LD	the majority of people has not been bitten by a tick	most of the people have been bitten by a tick or noticed a tick on their body once.
Knowledge of ticks / LD	widespread; some people know nothing, some people know a lot	medium to high

### Parents

#### Overview

Checking for ticks is determined by previous experience with ticks and LD (Table 2). Children of parents that check spend more time in high-endemic areas.

#### Knowledge of Ticks and LD

Parents that do not check had little knowledge of ticks and LD but thought that LD was a dangerous disease. The forest was the main location where they thought a child could be bitten. According to them, not all tick bites lead to LD. They did not know the first signs of LD or how fast a tick should be removed. Parents that check had medium knowledge of ticks and LD. They knew what a tick was and that they are very small. In addition, all of them knew about LD and thought it is a serious condition. All participants thought one could be bitten in a forest or in places with shrubs or grass. It was not clear to them that ticks also reside in dunes. None of the interviewees thought a tick bite always leads to LD. Most of them thought a tick should be removed within 24 hours after being bitten. Parents that check knew that EM occurs after an LD infection. A few also mentioned flu-like symptoms as a first sign of LD.

#### Experience With Ticks

Parents that do not check their children for tick bites had no experience with ticks or LD, while all of the parents that check their children for tick bites had previous experience. The latter were once bitten themselves, had one or more children or pets were bitten at least once, or they knew someone with LD.

#### Dealing With Tick Bites

Parents that do not check their children for tick bites have two strategies for removing ticks: using (tick) pliers or visiting their GP. They were unsure about their own abilities to remove ticks because they were afraid they could not remove the tick in its entirety. Most parents that check would remove a tick themselves if one of their children were bitten. However, they anticipated difficulties in the removal. They were afraid they would do it wrong, because they received different kinds of instructions (twisting when removing, pulling upward), or they were afraid that they would also remove the skin or would leave (a part of) the tick’s head behind.

I think I will have it removed by the family doctor. I don’t mind with my own cat… but, in the case of my own child, I want to have it done properly. It’s not that I won’t dare to do it, but I think I will notify the family doctor or will discuss with him what to do. I have one of those tick pliers. But, the last time the cat had a tick, it didn’t go as I wanted it to. So, I would go to the family doctor.

Woman; age, 32 years

#### Preventive Measures

All parents agreed about the issue of tick bite prevention. They knew they could prevent their children from being bitten by a tick by wearing the right kind of clothing. When prompted about the different precautions they could take, parents were not enthusiastic. They did not want to keep their child on paths in forests or parks, because they thought the children should be allowed to run freely. Wearing clothes that cover the body was not seen as a practical option when it was warm outside. Wearing a cap was not seen as an option, because the parents expected their child to remove it. Parents disliked the use of insect repellent sprays, because they contain diethyltoluamide (DEET), or the parents did not want to spray in their child or their clothes every day. Parents that do not check their children for tick bites do so because they think the chance of their child being bitten by a tick is very small or expect their child to notice a tick bite; they assumed that the children would “feel it.” Parents that do check their children do this when they thought their child was at risk of tick bite, for example, by having spent time in the forest. When they check their children, they mostly look at their armpits, neck, ears, groin, back of the knee, or “warm creases.”

#### Information Seeking Behavior

Parents that do not check indicated that they would search the Internet when they wanted information on how to prevent tick bites or how to remove ticks. Parents that do check expected the national government or the MHS to inform them about preventing tick bites and LD. When they need information on how to prevent tick bites, they would perform an Internet search using Google. To find information on how to remove ticks, they would perform a Google search or would consult their GP.

I think I would go to the family doctor if I don’t trust the situation [a child with a tick bite]. I would Google. That would be the way for me. Just type in “tick bite” and see where it gets me.

Woman; age, 47 years

All children of the parents we interviewed went into high-endemic areas with clubs or schools. Both groups of parents thought that checking for tick bites was their own responsibility when possible although they would like to be reminded to do so. When children are away overnight, the parents thought that it was the responsibility of the club or school to control for tick bites.

**Table 2:** Characteristics of parents and their children.

	Parents that do not check	Parents that check
Age	30 to 50 years old	30 to 50 years old
Family situation	married; 1 to 3 children	married; 1 to 3 children
Education	widespread	widespread
Pet ownership	low	about half of them has a pet
Direct experience with ticks/Lyme	none	all parents have experience with ticks or LD, either being bitten themselves, via a child, or via a pet
Knowledge of ticks/Lyme	low	medium

## Discussion

### Overview

In this paper, we have shown a lightweight qualitative approach by identifying risk groups and conducting in-depth interviews to create risk group profiles that can be used as inputs for targeting health communication. Such health communication is geared toward the characteristics and contexts of specific groups within a population and is generally more effective than one-size-fits-all communication [1]. To our knowledge, this is the first study in which this approach has been used providing information about tick bites and LD to the general Dutch population.

### Identifying Risk Groups

We identified four groups at a risk of being bitten by ticks bites and developing LD among the general Dutch population. The four groups were as follows: (1) outdoor people that check for tick bites, (2) outdoor people that do not check for tick bites, (3) parents that check their children for tick bites, (4) and parents that do not check their children for tick bites. Previous experience with tick bites or LD appeared to be the main denominator between the groups. Herrington [28] also identified previous experience with tick bites as one of the main factors governing compliance with preventive measures. The willingness to adopt measures that prevent tick bites (eg, wearing protective clothing) was low for all risk groups.

### Communicating Targeted Precautions

Our results were consistent with those reported by Gould et al [29], Marcu et al [7], and Beaujean et al [8], in that we found that checking for tick bites is a measure that is more easily adopted than preventing tick bites. Therefore, for all groups, the advice should primarily stress the importance of checking for tick bites. Moreover, we identified differences among groups, and therefore, health organizations should shift their focus from communicating expert-driven guidelines (promoting all precautions that can help) to communicating targeted precautions (those that members of a risk group are likely to adopt and/or fit with their perceptions). Using the profile of each risk group (Tables 1 and 2) and the motivations behind their behavior, health organizations can attune their informative and persuasive communication efforts. For example, outdoor people that do not check need to be educated about ticks and LD from scratch; they must be encouraged to check for tick bites after visiting a risk area; and they must be encouraged to remove ticks themselves instead of visiting their GP. However, outdoor people that do check already know the basics about ticks and LD and will remove ticks. These people should be provided with detailed information about the habitat of ticks. An approach similar to that mentioned above should be used in the case of parents. Parents that do not check their children for tick bites should be educated about the topic and should be motivated to check their children for tick bites and to independently remove the ticks that have bitten their children. Parents that do check are more willing to remove ticks that have bitten their child themselves but are afraid to do this wrong. Therefore, they do not need to be persuaded to remove the tick, but they require appropriate instructions.

### A Targeted Communication Strategy

Currently, we are developing a mobile app for ticks and LD as an example of a targeted communication strategy using a multidisciplinary requirements development approach [30]. We are using the profiles of each risk group (Tables 1 and 2) and the motivations underlying the behavior of the members of each risk group to determine the features and content of the app. A mobile app provides real-time up-to-date instructions and information and can be targeted to specific groups. Van Velsen et al showed that users need a video with information on how to remove a tick, a tick radar that indicates the actual tick activity on the basis of location and seasonality, and an alert that reminds people to check for tick bites at the end of the day when they have been in an endemic area. Finally, users expressed the need to document tick bites. A mobile app can provide these requirements identified by the users [31]. The app will offer only the selected information that is required according to the risk group. For example, for people that do not check (themselves or their children), information encouraging these people to check for tick bites after visiting a risk area will be included in the app. For people that do check, instructions on how to properly remove ticks will be added. Thus, the communication about ticks and LD will be targeted to great extent. Further studies are required to determine whether this method of communication is a more fruitful approach than communicating a one-size-fits-all message.

### Limitations

The main limitation of our study is the small sample size inherent to qualitative research and the approach used. Our study is a first step in identifying risk groups and understanding the differences between the risk groups used for the development of an intervention for the prevention of tick bites and LD. A larger number of respondents are required to draw more definite conclusions, for example, about the size of certain risk groups. However, the aim of this study and of creating a basis for targeted health communication is not to quantify conclusions (eg, give a certain percentage) that hold for a total population (and for which a quantitative study with a large sample would be most suited) but to identify risk groups, and most importantly, to understand the differences between the risk groups. Because we reached the point of data saturation, we were confident that we understand the differences between the risk groups (without actually quantifying these risk groups, which was not the aim of this study). Furthermore, we have presented an approach toward targeting health communication for situations in which access to large sets of data about a population is not available. When one wants to understand people’s behavior, or when large sets of data about a population are not available (as is often the case in public health), qualitative research is the only feasible means to elicit the necessary information (with limited budgets).

Another limitation relates to the defined risk groups. The distinction between outdoor people and parents with children might be somewhat confusing because some people might belong to both, for example, outdoor people with children. For these people, both targeted strategies would apply; one strategy for checking themselves and one strategy for checking their children. Although children can also be outdoor people, they do not belong to the outdoors group because they cannot be held responsible for checking themselves for ticks; this is the responsibility of parents.

### Conclusions

Finally, the extreme form of targeting is tailoring, that is, tuning health communication toward the characteristics and context of the individual. This holds the potential to increase a person’s attention or motivation about a specific health issue or healthy behavior [32,33]. Further, with the development of new technologies in the last decade, health organizations have gained a wide range of possibilities for gearing messages toward the individual user. However, although often effective [34], tailoring is not always preferred over targeting, because this choice depends on the complexity of the targeted behavior, the available budget, the variability of behavioral determinants among individuals, and the availability of mechanisms for assessing an individual’s characteristics and context [35]. Moreover, in many cases, a combination of a targeted and tailored approach is the optimal health communication strategy [35]. Therefore, identifying differences among risk groups is and remains an important facet of tailoring health advice be it toward groups or individuals. The lightweight qualitative approach presented in this paper is highly relevant in achieving this objective.

## Multimedia Appendix 1

Interview guide.

## Abbreviations

COPD: chronic obstructive pulmonary disease
DEET: diethyltoluamide
EM: erythema migrans
GP: general practitioner
HIV: human immunodeficiency virus
LD: Lyme disease
MHS: municipal health services
