# Supplementary material for: Using Risk Group Profiles as a Lightweight Qualitative Approach for Intervention Development: An Example of Prevention of Tick Bites and Lyme Disease
Source: JMIR Res Protoc. 2013 Oct 30;2(2):e45. doi: 10.2196/resprot.2760 (PMC3841373; doi:10.2196/resprot.2760)
Supplement: Supplementary file 1 [file resprot_v2i2e45_app1.pdf]

Appendix A

Interview Guide for Outdoor people

Start of the interview
Each interview started with a short introduction. Interviewees were asked for permission to audiotape the interview.

Demographics

1 Gender
2 Age
3 Nationality
4 Do you speak/understand the Dutch language?
5 Family situation
6 Profession
7 Education

Knowlegde of ticks and Lyme Disease

8 Do you know what a tick is?
9 Do you know what Lyme Disease is?
10 How do you get Lyme Disease?
11 Does a tick bite always leads to Lyme Disease?
12 How fast should you remove a tick?
13 How do you know you have Lyme Disease?
14 Where in nature can you get a tick bite?

Experience with tick bites and Lyme Disease

15 Have you ever seen a tick?
16 Where did you first hear on ticks and Lyme Disease?
17 Have you ever been bitten by a tick?
18 What barriers do you expect when searching for information on tick bites and Lyme Disease?
19 Who do you expect to inform you on tick bites and Lyme Disease?
20 What do you think about the current information on tick bites and Lyme Disease?
21 What do you want to know in order to handle tick bites better?
22 How do you feel about the following organizations who can inform you on tick bites and Lyme Disease? Are these the correct organizations?

- The regional Municipal Health Service
- The National Insitute for Public Health and the Environment
- The municipality
- Intermediars (sport clubs, outdoor clubs)

- General Practitioner
- National Lyme organization

Perception and behavior regarding preventive measures

23 How do you prevent yourself from getting a tick bite?

24 What would you do in case of a tick bite?

25 How severe do you perceive Lyme Disease?

26 What problems do you expect when getting a tick bite or treating a tick bite?

Frequency of visits to high-risk areas

27 How often do you visit:

- Your backyard or other people's backyard
- Forrest
- Heath land
- Dunes
- City parks

Attitude towards checking yourself for tick bites

28 Do you ever check yourself for tick bites? When?

29 How do you feel about the following preventive measures against tick bites:

- Stay on the paths in forests or parks
- Wearing long pants, putting you pant legs in your soks, wearing closed shoes
- Using insect repellent for uncovered skin
- Using DEET on your clothes

Knowlegde and use of mobile technology

30 Do you easily adopt new technologies?

31 Do you like to use new technologies, like internet and mobile phones?

32 Do you know what a smartphone is?

33 Do you own a smartphone?

34 Do you know what to do with a QR code?

Tick and Lyme Disease related information seeking behavior

35 How would the ideal website on tick bites and Lyme Disease look like?

36 How would the ideal smartphone application on tick bites and Lyme Disease look like?

37 Do you feel comfortable getting information on tick bites and Lyme Disease from a website?

38 Would you use this website and why?

39 Do you feel comfortable getting information on tick bites and Lyme Disease for a mobile application?

40 Would you install this mobile application on your smartphone?

41 What other ways do you suggest to get more information on tick bites and Lyme Disease?

End of the interview

42 Do you have any questions?

Interview Guide for Parents

Start of the interview
Each interview started with a short introduction. Interviewees were asked for permission to audiotape the interview.

Demographics

1 Gender
2 Age
3 Nationality
4 Do you speak/understand the Dutch language?
5 Family situation
6 How old are your children?
6 Profession
7 Education

Knowlegde of ticks and Lyme Disease

8 Do you know what a tick is?
9 Do you know what Lyme Disease is?
10 How do you get Lyme Disease?
11 Does a tick bite always leads to Lyme Disease?
12 How fast should you remove a tick?
13 How do you know you have Lyme Disease?
14 Where in nature can a child get a tick bite?

Experiences with tick bites and Lyme Disease

15 Have you ever seen a tick?
16 Where did you first hear on ticks and Lyme Disease?
17 Has one of you children ever been bitten by a tick?
18 What barriers do you expect when searching for information on tick bites and Lyme Disease?
19 Who do you expect to inform you on tick bites and Lyme Disease? Why?
20 What do you think about the current information on tick bites and Lyme Disease?
21 What do you want to know in order to handle tick bites better?
22 How do you feel about the following organizations who can inform you on tick bites and Lyme Disease? Are these the correct organizations?

- The regional Municipal Health Service
- The National Insitute for Public Health and the Environment
- The municipality
- Intermediars (sport clubs, outdoor clubs)
- General Practitioner

- National Lyme organization

Perception and behavior regarding preventive measures

23 How do you prevent your children from getting a tick bite?

24 What would you do in case of a tick bite?

25 How severe do you perceive Lyme Disease?

26 What problems do you expect when getting a tick bite or treating a tick bite?

Frequency of visits to high-risk areas

27 How often do your children visit:

- Your backyard or other people's backyard
- Forrest
- Heath land
- Dunes
- City parks

28 Do you children attend camps or school trips at/to the forest?
If yes, do you think it is important your children will be checked for tick bites?
Who should be responsible for this?

Attitude towards checking your child for tick bites

29 Do you ever check your children for tick bites? When?

30 How do you feel about the following preventive measures against tick bites:

- Stay on the paths in forests or parks
- Wearing long pants, putting you pant legs in your soks, wearing closed shoes
- Using insect repellent for uncovered skin
- Using DEET on your children’s clothes
- Let your child wear a cap

Knowlegde and use of mobile technology

31 Do you easily adopt new technologies?

32 Do you like to use new technologies, like internet and mobile phones?

33 Do you know what a smartphone is?

34 Do you own a smartphone?

35 Do you know what to do with a QR code?

Tick and Lyme Disease related information seeking behavior

36 How would the ideal website on tick bites and Lyme Disease look like?

37 How would the ideal smartphone application on tick bites and Lyme Disease look like?

38 Do you feel comfortable getting information on tick bites and Lyme Disease from a website?

39 Would you use this website and why?

40 Do you feel comfortable getting information on tick bites and Lyme Disease for a mobile application?

41 Would you install this mobile application on your smartphone?

42 What other ways do you suggest to get more information on tick bites and Lyme Disease?

End of the interview

43 Do you have any questions?
